# Short echo time first-echo images of T2* mapping for detecting structural sacroiliac joint lesions in axial spondyloarthritis: a CT-referenced study

**DOI:** 10.3389/fimmu.2026.1946946

**Published:** 2026-10-22

**Authors:** İdil Kurut Aysin, Murat Yoğurtçu, Atilla Hikmet Çilengir, Hasan Kocaayan, Ayten Özkan, Özge Yoğurtçu, Yiğit Atli, Mustafa Özmen, Dilek Solmaz, Özgür Tosun, Servet Akar

**Affiliations:** 1 Department of Rheumatology, Balıkesir Ataturk City Hospital, Balikesir, Türkiye; 2 Department of Radiology, Tire State Hospital, Izmir, Türkiye; 3 Department of Radiology, Faculty of Medicine, Izmir Democracy University, Izmir, Türkiye; 4 Department of Rheumatology, Izmir Katip Celebi University Ataturk Training and Research Hospital, Izmir, Türkiye; 5 Department of Radiology, Izmir Katip Celebi University Ataturk Training and Research Hospital, Izmir, Türkiye

**Keywords:** axial spondyloarthritis, magnetic resonance imaging, sacroiliac joint, sacroiliitis, structural lesions, T2* mapping

## Abstract

**Objective:**

To assess the CT-referenced diagnostic performance of short-echo time (TE) first-echo images derived from a T2* mapping acquisition for detecting sacroiliac joint (SIJ) structural lesions in patients evaluated for axial spondyloarthritis (axSpA), compared with conventional T1-weighted turbo spin-echo (T1-TSE) MRI.

**Methods:**

In this study, 54 patients underwent SIJ CT and 3T MRI; 52 were included in the final analysis. MRI included T1-TSE and a T2* mapping acquisition; the first-echo magnitude images (TE = 4.36 ms) were used for structural assessment. Two blinded readers scored CT and MRI using the Berlin and SPARCC structural scoring systems. Patient-level sensitivity (SE), specificity (SP), and diagnostic accuracy (DA) were calculated using CT as the reference. Correlation with CT was assessed using Pearson’s r, and inter-rater agreement was evaluated (κ for Berlin binary positivity; ICC for continuous sum scores).

**Results:**

Forty-three of 52 patients fulfilled ASAS 2009/2011 spondyloarthritis classification criteria. Compared with T1-TSE, short TE-T2* images showed higher diagnostic performance for erosions (SE 88.6% vs 51.4%; SP 70.6% vs 82.4%; DA 82.6% vs 61.7%) and sclerosis (SE 86.2% vs 34.5%; SP 95.7% vs 100%; DA 90.3% vs 62.4%), while both sequences achieved high accuracy for joint space changes (DA 98.2%; T2*: SE/SP 98%/100%; T1: 100%/66.7%). Agreement with CT was stronger for short TE-T2* than for T1-TSE for Berlin erosion and sclerosis sum scores (r=0.913 and 0.921 vs 0.646 and 0.758), with substantial reader agreement for erosion and sclerosis positivity (κ=0.805 and 0.731 vs 0.564 and 0.598).

**Conclusion:**

Short TE first echo images from a T2* mapping acquisition provide an accessible, radiation-free, erosion-sensitive approach that demonstrates higher CT-referenced detection of erosions and sclerosis compared with conventional T1-TSE imaging, with strong CT agreement and robust inter-rater reliability.

## 1 Introduction

Spondyloarthritis (SpA) is a heterogeneous group of inflammatory rheumatic diseases characterized by shared clinical, laboratory, and imaging features that involve the axial skeleton and/or peripheral joints (1). According to the Assessment of SpondyloArthritis International Society (ASAS) classification criteria SpA is categorized into axial (axSpA) and peripheral (pSpA) disease (2, 3). AxSpA is further subclassified as radiographic axSpA (r-axSpA; ankylosing spondylitis) or non-radiographic axSpA (nr-axSpA) based on the presence or absence of definite radiographic sacroiliitis.

Although the ASAS classification criteria define a positive magnetic resonance imaging (MRI) primarily by the presence of inflammatory lesions, namely subchondral bone marrow edema, concerns have been raised regarding the specificity of this finding. Bone marrow edema can frequently occur in individuals performing heavy physical labor, elite athletes, and especially women in the postpartum period. Recent studies have demonstrated that the combined assessment of structural changes alongside bone marrow edema on MRI might improve the diagnostic accuracy of axial SpA and provide additional value in differentiating patients with non-specific bone marrow edema from those with axSpA (4–10).

Computed tomography (CT) is considered the gold standard for the assessment of sacroiliac joint (SIJ) structural lesions due to its superior ability to visualize calcification- and ossification-related changes within bone (11, 12). However, the associated exposure to ionizing radiation limits its repeated use, particularly for longitudinal monitoring of structural damage progression in axSpA (13, 14). Moreover, conventional CT cannot visualize active inflammatory findings, such as bone marrow edema, capsulitis, synovitis, or enthesitis which are readily detectable on MRI. Therefore, achieving CT-like visualization of structural bone lesions with MRI has become a highly desirable goal in the imaging assessment of axSpA.

Among MRI sequences, the T1-weighted turbo spin-echo (T1-TSE) is the most commonly used sequence in clinical practice for detecting structural lesions. Nevertheless, its diagnostic accuracy is limited, which has prompted the development and evaluation of alternative MRI sequences that can provide improved visualization of structural changes in particular erosions (15, 16).

The ASAS-SPARTAN collaboration also recommends the inclusion of erosion-sensitive sequences in the standard SIJ image acquisition protocol, including multiple thin-slice 3D gradient-echo–based techniques such as Liver Acquisition with Volume Acceleration (LAVA), sometimes referred to as Black Bone imaging, susceptibility-weighted imaging (SWI), zero echo time (ZTE), ultrashort echo time (UTE), and volumetric interpolated breath-hold examination (VIBE) sequences (17). However, because these erosion-sensitive sequences require ultrashort echo times, thin-slice 3D acquisitions, synthetic CT reconstruction, or specialized post-processing, they cannot be optimized on every MRI system, which limits their widespread use. In addition, they increase MRI examination time and require additional time for post-processing.

T2*-weighted gradient-echo MRI is highly sensitive to local magnetic field inhomogeneities caused by both paramagnetic substances (such as iron and deoxyhemoglobin) and diamagnetic materials (such as calcifications). These susceptibility differences accelerate signal dephasing and lead to signal loss on T2*-weighted gradient-echo images, producing hypointense regions that correspond to local field distortion. Consequently, microbleeds, iron deposition, air–tissue interfaces, calcifications, and cortical bone (high calcium content) can be delineated on T2*-weighted images (18, 19).

In T2*-weighted imaging, signal intensity decays as a function of the echo time (TE), with tissues exhibiting short T2* values, such as cortical bone, ligaments, and calcified cartilage, losing signal more rapidly than long-T2* tissues like fat or muscle. Therefore, on images acquired at shorter TE values, susceptibility-related signal loss in the tissues adjacent to bone is limited, and cortical bone is depicted as a sharply delineated signal void, which may improve visualization of the cortical margin (20). The contrast mechanism of gradient-echo imaging, in which healthy cortical bone appears dark due to calcium-related signal loss and erosion cavities appear relatively bright, has been recognized for nearly three decades. Early studies used short-echo-time gradient-echo imaging (i.e., T2*-weighted gradient-echo) to evaluate the SIJ (21). However, the diagnostic performance of this approach for detecting structural SIJ lesions in axial spondyloarthritis has not been systematically investigated.

Despite the advantages of T2*-weighted MRI sequences in visualizing cortical bone and consequently, erosions, there remains a knowledge gap in the literature regarding the evaluation of structural lesions on SIJ. To our knowledge, this is the first study to evaluate a multi-echo T2* mapping acquisition using short-TE first-echo images for detecting structural lesions in the SIJ, with CT as the reference standard and also using both the Berlin and SPARCC scoring systems.

In this study, we assessed the performance of a multi-echo T2* mapping sequence for detecting structural lesions of the SIJ, using CT as the reference standard. Specifically, we analyzed the first-echo magnitude images (TE = 4.36 ms), in which the short TE limits susceptibility-related signal loss and allows sharp delineation of cortical bone, and compared them with conventional T1-TSE imaging.

## 2 Materials and methods

### 2.1 Patients

We prospectively included 54 patients with either a previous diagnosis of axSpA or clinical suspicion of axSpA. Clinical suspicion of axSpA was based on chronic back pain with onset before the age of 45 years and the presence of at least one SpA feature, including inflammatory back pain, arthritis, enthesitis (heel), uveitis, dactylitis, psoriasis, Crohn’s disease/ulcerative colitis, a good response to NSAIDs, a family history of SpA, HLA-B27 positivity, or elevated CRP. Patients with an established diagnosis of axSpA were referred for imaging to evaluate disease activity. Following clinical evaluation by a senior rheumatologist, patients were classified as r-axSpA, nr-axSpA, or peripheral SpA according to the ASAS classification criteria, while those who did not fulfill the ASAS criteria and had no evidence of inflammatory rheumatic disease were classified as non-inflammatory. Exclusion criteria included contraindications to MRI (e.g., pacemaker or other metallic implants), claustrophobia, and pregnancy.

The study protocol including the CT examination performed as the reference standard, was approved by the İzmir Katip Çelebi University Clinical Research Ethics Committee (date: 11.05.2023 Decision No. 29) The study workflow, including patient enrollment, imaging protocol, image analysis, and statistical evaluation, is summarized in **Figure 1**.

**Figure 1:**
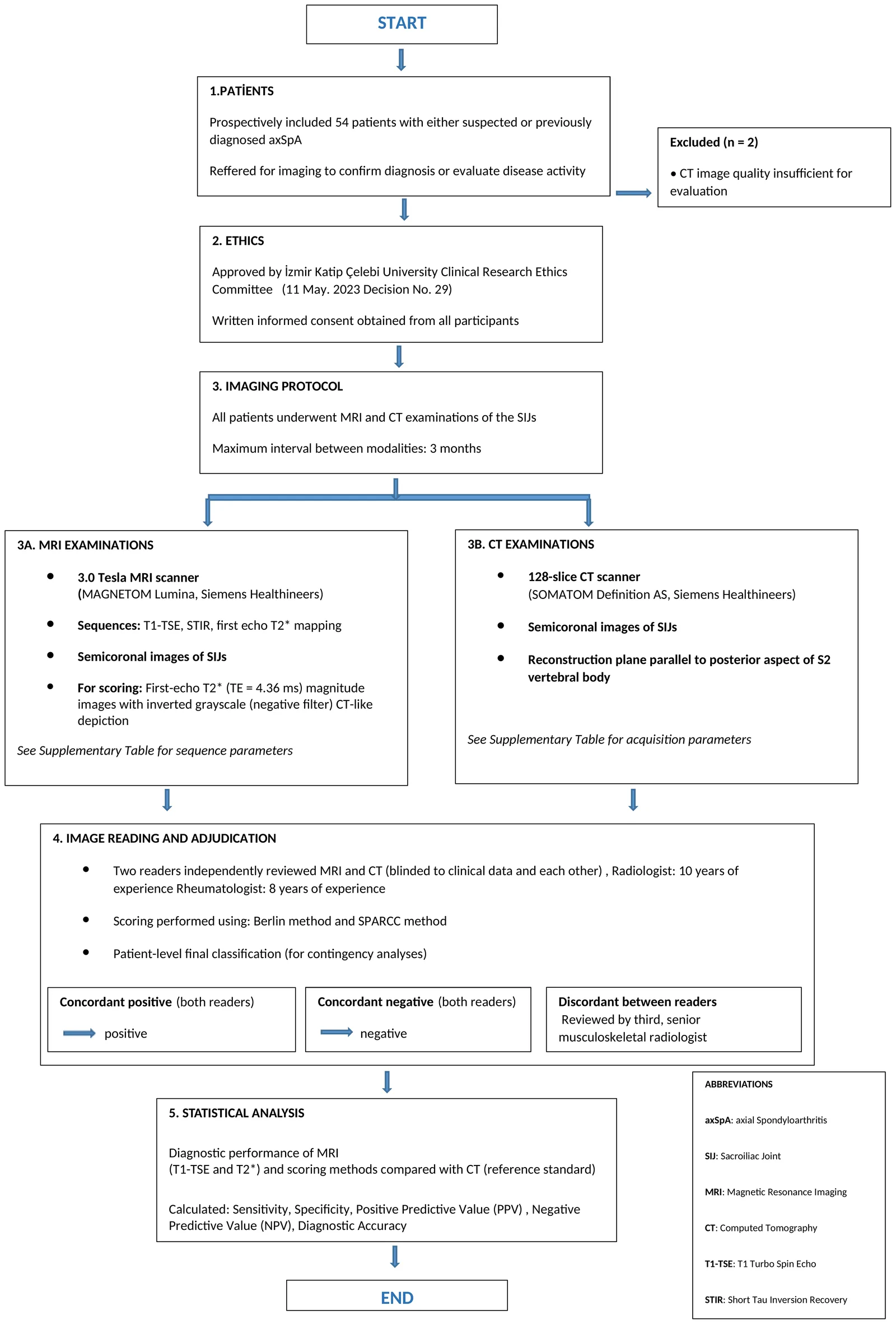
Flow diagram of the study design.

### 2.2 Imaging protocol

All enrolled patients underwent MRI and CT examinations of the SIJs, with a maximum interval of 3 months between modalities. MRI scans were performed according to a standardized imaging protocol that included T1-TSE, short tau inversion recovery (STIR), and multi-echo T2* mapping sequence.

#### 2.2.1 MRI examinations

Semicoronal images of the SIJs were obtained in all patients using the same 3.0 Tesla MRI scanner (MAGNETOM Lumina, Siemens Healthineers, Erlangen, Germany). The T1-TSE sequence was acquired according to established protocols described in the literature (22). Additionally, T2* mapping and STIR images were acquired for further tissue characterization. MRI sequence parameters are presented in **Supplementary Table 1**.

The T2* mapping acquisition was the vendor-provided multi-echo spoiled gradient-echo protocol, used with its default parameters (**Supplementary Table 1**). For structural lesion assessment, the use of magnitude images from the first echo (TE = 4.36 ms) was pre-specified before image analysis, as the shortest TE minimizes susceptibility-related signal loss and blooming, so that cortical and sclerotic bone are depicted as a sharply delineated signal void against the adjacent marrow and joint space. With this short TE and the flip angle used, the first-echo images have mixed contrast, including proton-density/T1 and T2* contributions, rather than pure T2* weighting; they were used solely for morphological assessment, and no quantitative T2* values were analyzed … For visual scoring, first-echo T2* images were reviewed using an inverted grayscale (negative filter) to provide a CT-like depiction of cortical margins (**Figure 2**).

**Figure 2:**
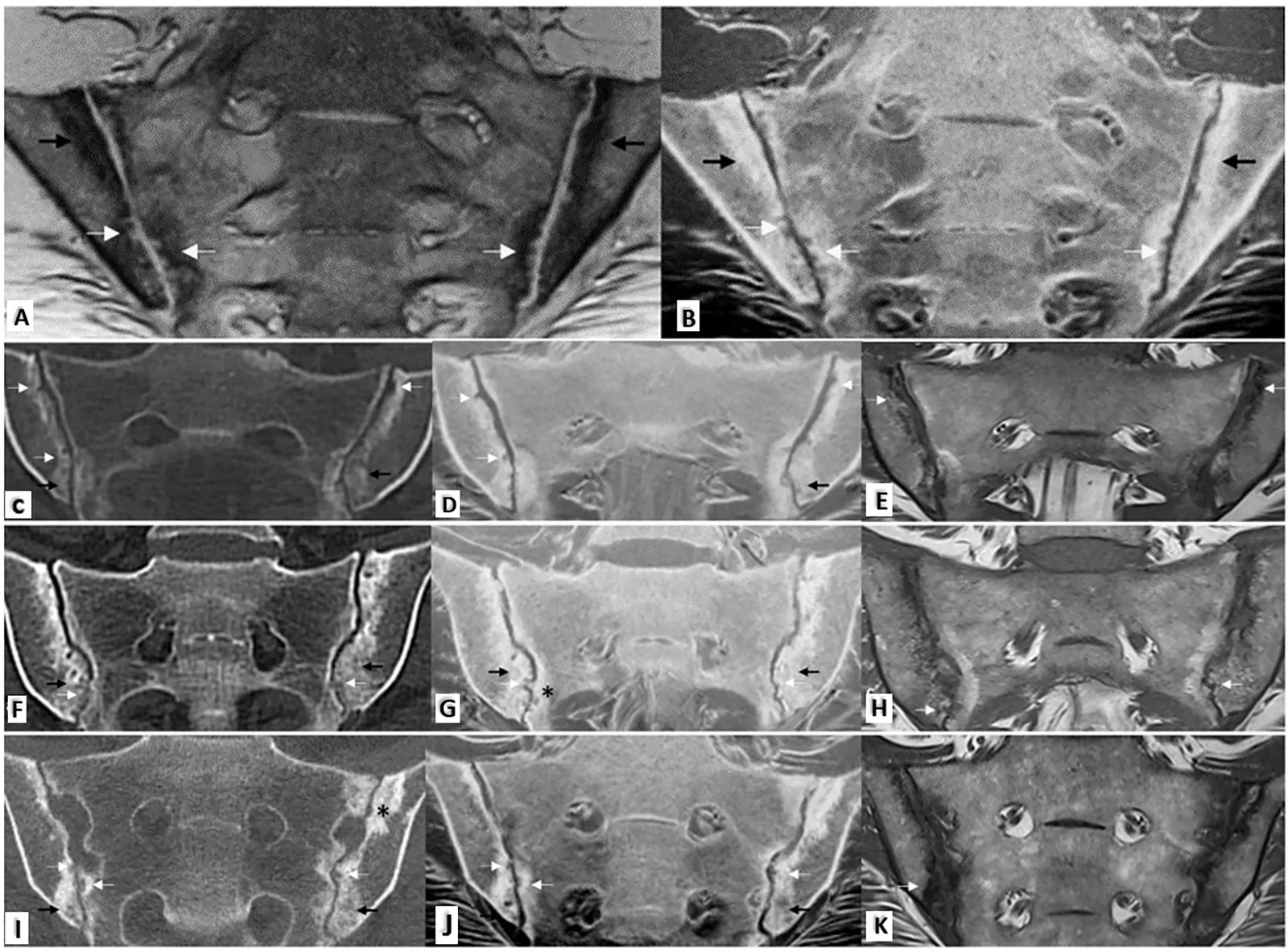
Short-TE first-echo T2* image appearance and representative examples of structural sacroiliac joint lesions on CT, short-TE first-echo T2* MRI, and T1-TSE MRI. **(A)** Original short-TE first-echo T2* magnitude image and **(B)** the corresponding CT-like image displayed using inverted grayscale (negative filtering). Multiple erosions (white arrows) and areas of subchondral sclerosis (black arrows) are visible in both sacroiliac joints. In the lower three rows, CT, inverted short-TE first-echo T2* MRI, and T1-TSE MRI are shown from left to right, respectively. **(C–E)** Concordant depiction across modalities. CT **(C)**, short-TE first-echo T2* MRI **(D)**, and T1-TSE MRI **(E)** demonstrate multiple bilateral erosions (white arrows). On T1-TSE MRI, sclerosis and backfill partly obscure the erosions. Consistent with CT, short-TE first-echo T2* MRI depicts more extensive bilateral iliac subchondral sclerosis (black arrows) than T1-TSE MRI. **(F–H)** higher lesion conspicuity on short-TE T2* MRI. CT **(F)**, short-TE T2* MRI **(G)**, and T1-TSE MRI **(H)** show multiple erosions (white arrows) in both sacroiliac joints. Erosions in the inferior quadrant of the right ilium are well depicted on CT and short-TE T2* MRI, but are poorly visualized on T1-TSE MRI. Compared with CT, the visible erosions are better appreciated on short-TE T2* MRI. In addition, a non-bridging bone bud in the inferior quadrant of the right sacrum (asterisk), together with the underlying erosion, is more clearly visualized on the short-TE T2* MRI slice. Short-TE T2* MRI also shows more extensive bilateral iliac subchondral sclerosis (black arrows), consistent with CT. **(I–K)** Greater lesion conspicuity on CT. CT **(I)**, short-TE first-echo T2* MRI **(J)**, and T1-TSE MRI **(K)** demonstrate multiple bilateral erosions (white arrows) and areas of sclerosis (black arrows). Bilateral erosions are more clearly depicted on CT. A subtle erosion in the superior quadrant of the left ilium (asterisk) is visible on CT but not on the corresponding MRI section. On T1-TSE MRI, sclerosis and backfill partly obscure the erosions.

#### 2.2.2 CT examinations

Semicoronal images of the SIJs were obtained in all patients. CT scans of the SIJs were performed using a 128-slice scanner (*SOMATOM Definition AS*, Siemens Healthineers, Erlangen, Germany). The CT acquisition parameters are presented in **Supplementary Table 2**, and radiation dose metrics are summarized in **Supplementary Table 3**. Automatic exposure control was used. The effective dose was estimated from the dose -length product using an ICRP 103-based conversion coefficient (23). Semicoronal images were reconstructed in a plane parallel to the posterior aspect of the S2 vertebral body, which served as the reference plane.

#### 2.2.3 Imaging score

The MRI and CT images were independently reviewed by two readers (a radiologist with 10 years of experience and a rheumatologist with 8 years of experience) who were blinded to all clinical data and the findings of the other imaging modality. To minimize recall bias, image interpretation was performed in a predefined sequential order. T1-TSE images were evaluated first, followed by first-echo T2* images after a 3-week washout interval; cases were presented in randomized order during both MRI assessments. CT images were evaluated one month after completion of all MRI assessments. Structural lesions were scored using the Berlin method (24) and the Spondyloarthritis Research Consortium of Canada (SPARCC) scoring system (25). Before final reading session, both readers underwent a calibration session using images from 10 patients who were not included in the final study cohort. These cases were independently scored by the two readers and subsequently reviewed together with the third reader to establish consensus on the interpretation and scoring of structural lesions according to the predefined scoring criteria.

For patient-level contingency analyses, the final binary classification was determined by an adjudicated majority rule. Concordant positive ratings by both primary readers were accepted as positive; concordant negative ratings were accepted as negative; and cases with discordant ratings were reviewed by a third, senior musculoskeletal radiologist. The final classification was based on the agreement of at least two of the three readers. These final adjudicated classifications were used to calculate sensitivity, specificity, positive predictive value, negative predictive value, and diagnostic accuracy, with CT serving as the reference standard.

##### 2.2.3.1 Berlin scoring method

Each SIJ was assessed at three predefined semicoronal positions (anterior, middle, and posterior) as described by Diekhoff et al. (24). For each position, the joint was divided into four quadrants (upper and lower sacral and iliac regions), yielding 12 regions per SIJ and 24 per patient.

The ventral (anterior) position corresponded to slices in which no sacral neural foramina were visible. The middle position corresponded to slices in which the sacral foramina were depicted. The dorsal (posterior) position corresponded to slices in which sacral nerve roots and the spinal canal were visible. Lesion definitions were as follows:

- Erosion: On CT, a complete loss of the subchondral cortical bone within the defined area, appearing as a hypodense defect; on T1-TSE MRI, this corresponded to a focal area of low signal intensity within the subchondral bone. On first-echo T2* images reviewed with an inverted grayscale, erosions were identified using the same CT-analogous morphologic criterion of cortical interruption.
- Sclerosis: On CT, a hyperdense subchondral lesion measuring ≥5 mm in depth perpendicular to the joint surface, clearly denser than the surrounding trabecular bone; on T1-TSE MRI, represented by areas of very low signal intensity. On first-echo T2* images reviewed with an inverted grayscale, sclerosis was identified using the same CT-analogous morphologic criterion.
- Joint space changes: Defined as a change in the joint space width, including narrowing, widening, or complete disappearance of the joint space (ankylosis).
- Ankylosis: On CT, it is defined as a dense bony structure bridging the joint space; on T1-TSE MRI, it is characterized by the continuity of high bone marrow signal intensity across the joint space. On first-echo T2* images reviewed with an inverted grayscale, ankylosis was identified using the same CT-analogous morphologic criterion.

Each lesion type was scored separately for all regions:

- Erosions: scored 0–3 per region, total possible score 0–72.
- Sclerosis: scored 0–2 per region, total possible score 0–48.
- Joint space changes: scored 0–4 per region, total possible score 0–48.

##### 2.2.3.2 SPARCC scoring method

On semicoronal MRI slices through the cartilaginous portion of the SIJ, the transitional slice, defined as the first slice showing the ligamentous portion of the joint, and the four consecutive slices anterior to it were used for scoring, for a total of five slices.

Fat metaplasia and erosions were assessed in eight quadrants (right and left SIJ; upper and lower; sacral and iliac sides) of five slices, resulting in a maximum total score of 40 points. Backfill lesions and bony ankylosis were evaluated in four regions (right and left SIJ; upper and lower halves), with a maximum total score of 20 points (25). Because CT does not provide a reference standard for fat metaplasia or backfill, only the erosion and ankylosis components of the SPARCC structural score were included in the CT-referenced analyses.

##### 2.2.3.3 Definition of positive imaging findings

In the Berlin scoring system, a structural lesion (erosion, sclerosis, or joint space change) was considered unequivocally present if a score ≥2 was assigned in any of the 24 regions evaluated (12 per SIJ). For patient-level analyses, a patient was classified as lesion-positive if at least one region met this threshold. For statistical analyses based on lesion presence/absence, final lesion status was determined according to the adjudicated majority rule described above. In contrast, no binary patient-level positivity threshold was defined for the SPARCC structural scores, as no validated cut-off for patient-level positivity has been established.

### 2.3 Statistical analysis

The minimum required sample size was calculated *a priori* using G*Power version 3.1.9, based on previously reported differences in sensitivity for the detection of sacroiliac joint sclerosis between MRI sequences. Assuming an effect size of 0.5, an alpha error of 0.05, and 90% power, the minimum required sample size was 33 patients.

Demographic data were presented as mean ± standard deviation (SD) for normally distributed variables and as median (interquartile range, IQR) for non-normally distributed variables. For Berlin-based binary outcomes, a lesion was considered present at the patient level if a score ≥2 was recorded in at least one of the 24 evaluated regions; inter-rater agreement for these binary lesion classifications was quantified using Cohen’s kappa (κ). SPARCC structural outcomes were analyzed as only continuous sum scores, and inter-rater agreement was assessed using intraclass correlation coefficients (ICCs) with 95% confidence intervals. Intra-rater reliability was assessed by re-evaluating MRI and CT images of 10 randomly selected patients, and ICC was calculated using a two-way mixed-effects model (26). For correlations analyses, the sum scores assigned by the two readers for each structural lesion and imaging modality were averaged to obtain a mean sum score. Correlations between CT- and MRI-derived mean sum scores were assessed using Pearson’s correlation coefficient (r) (27). Diagnostic performance was evaluated only for Berlin-based binary outcomes at the patient level using sensitivity, specificity, positive predictive value, negative predictive value, and overall accuracy, with CT as the reference standard. A two-sided p-value <0.05 was considered statistically significant.

## 3 Results

### 3.1 Patients

The SIJ MRI and CT scans were acquired within 3 months in 54 patients who were either referred for evaluation of suspected axSpA or were already under follow-up with a confirmed diagnosis of SpA. The mean interval between MRI and CT examinations was 1.41 ± 2.09 weeks (median, 1 week; range, 1–12 weeks). Due to motion artifacts on CT in two female patients, 52 patients were included in the final analysis: 37 (71.2%) with r-axSpA, 5 (9.6%) with nr-axSpA, 1 (1.9%) with pSpA, and 9 (17.3%) with non-inflammatory conditions. Non-inflammatory conditions were lumbar disc herniation (n=2), SIJ dysfunction (n=2), osteitis condensans ilii (n=1), degenerative SIJ disease (n=3), and fibromyalgia (n=1).

The mean (± SD) age of the overall cohort was 43.2 ± 10.2 years, and 55.8% of the patients were male. Among patients with SpA (axSpA + pSpA; n = 43), BASDAI data were available for 42 patients, with a mean score of 4.94 ± 2.3 (scale, 0–10); CRP data were available for 41 patients, with a median concentration of 4.68 mg/dL (IQR, 8). The mean symptom duration for the SpA group was 15.0 ± 9.4 years (range, 3–44 years). Among patients with available HLA-B27 data, 11 of 37 (29.7%) were HLA-B27 positive. Among the 41 SpA patients with available clinical feature data, uveitis, psoriasis, and arthritis were present in 11 (26.8%), 2 (4.9%), and 3 (7.7%) patients, respectively. Detailed demographic and clinical characteristics of the study population are presented in **Table 1**.

**Table 1:** Demographic and clinical characteristics of the study population.

Characteristic	Value
Mean age, years ± SD (range)	43.2 ± 10.2 (23-68)
Male, *n/N (%)*	29/52 (55.8%)
HLA-B27 positivity, *n/N (%)*	11/37 (29.7%)
Median CRP, mg/dL (IQR)	4.68 (8)
Mean BASDAİ score (0-10) ± SD	4.94 ± 2.3
Mean symptom duration, years ± SD (range)	14.97 ± 9.44 (3-44)
Presence of uveitis, *n/N (%)*	11/41 (26.8%)
Presence of psoriasis, *n/N (%)*	2/41 (4.9%)
Presence of arthritis, *n/N (%)*	3/41 (7.7%)
*Clinical diagnosis*, *n/N (%)* Axial spondyloarthritis r-axSpA nr-axSpA Peripheral SpA Non-inflammatory disease	42/52 (80.8%) 37/52 (71.2%) 5/52 (9.6%) 1/52 (1.9%) 9/52 (17.3%)

SD, standard deviation; HLA-B27, human leukocyte antigen B27; CRP, C-reactive protein; IQR, interquartile range; BASDAI, Bath Ankylosing Spondylitis Disease Activity Index; r-axSpA, radiographic axial spondyloarthritis; nr-axSpA, non-radiographic axial spondyloarthritis; SpA, spondyloarthritis.

### 3.2 Erosions

Based on the Berlin scoring system, 35 patients were considered positive for erosions on CT, 21 on T1-TSE, and 36 on T2*.The mean total erosion score was 12.13 ± 8.13 for CT, 6.91 ± 3.63 for T1-TSE, and 13.54 ± 8.27 for T2*. Correlation of Berlin erosion scores with CT was strong for T1-TSE (*r* = 0.646) and very strong for T2* (*r* = 0.913). Inter-rater reliability was excellent for CT (ICC = 0.897, 95% CI: 0.820–0.941, *p* < 0.001), good for T1-TSE (ICC = 0.695, 95% CI: 0.474–0.823, *p* < 0.001), and excellent for T2* (ICC = 0.924, 95% CI: 0.868–0.956, *p* < 0.001).

According to the SPARCC erosion score, the mean total erosion score was 15.36 ± 8.79 on CT, 9.19 ± 4.43 on T1-TSE, and 17.13 ± 9.17 on T2*. The relationship between SPARCC erosion scores and CT was moderate for T1-TSE (*r* = 0.597) and very strong for T2* (*r* = 0.909).

For SPARCC erosion scores, inter-rater agreement was excellent for first-echo T2* images and CT (ICC = 0.953, 95% CI: 0.918–0.973 and ICC = 0.942, 95% CI: 0.899–0.967, respectively) and good for T1-TSE images (ICC = 0.785, 95% CI: 0.630–0.875).

The diagnostic accuracy for erosion detection, compared with CT, is illustrated in **Figure 3**.

**Figure 3:**
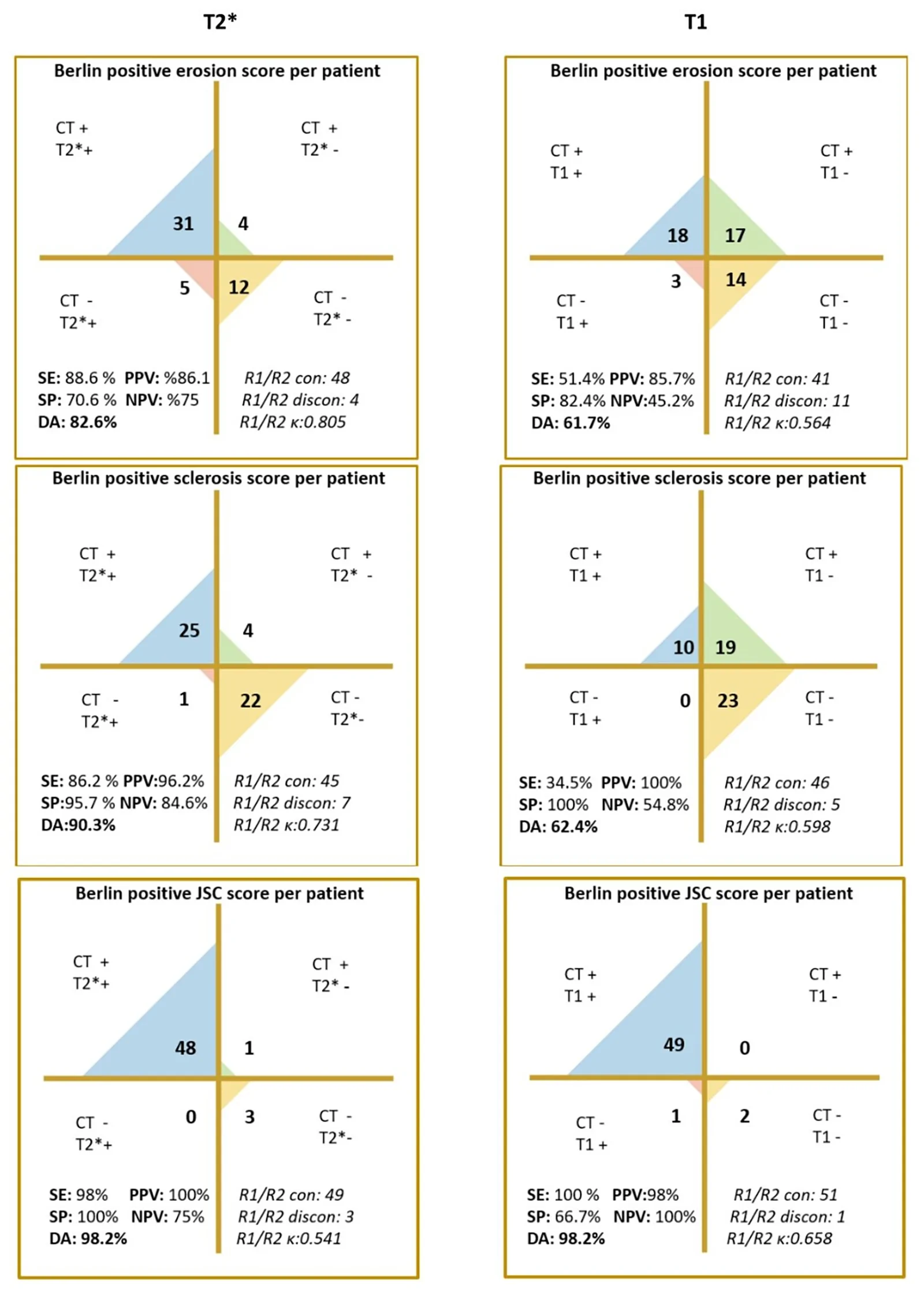
Contingency analysis of T1-TSE MRI and short-TE T2* MRI versus CT. For definitions of positive findings and scoring items, see the Methods section of the text. κ, Cohen’s κ for agreement of the two readers; SE, sensitivity; SP, specificity; PPV, positive predictive value; NPV, negative predictive value; using CT as the standard of reference.

### 3.3 Sclerosis

Sclerosis was identified on CT in 29 patients, on T1-TSE in 10, and on T2* in 26. The mean total sclerosis score was 11.01 ± 7.59 for CT, 3.71 ± 3.77 for T1-TSE, and 10.93 ± 8.99 for T2*.

In comparison with CT, the correlation of the Berlin sclerosis score was strong for T1-TSE (*r* = 0.758) and very strong for T2* (*r* = 0.921). Inter-rater reliability was excellent for CT (ICC = 0.939, 95% CI: 0.894–0.965, *p* < 0.001), excellent for T1-TSE (ICC = 0.882, 95% CI: 0.797–0.932, *p* < 0.001), and excellent for T2* (ICC = 0.930, 95% CI: 0.879–0.960, *p* < 0.001).

Diagnostic accuracy results for sclerosis are presented in **Figure 3**.

### 3.4 Joint space changes

Joint space changes (JSC) were observed in 49 patients on CT, 50 on T1-TSE, and 48 on T2*. The mean total JSC score was 21.04 ± 6.62 for CT, 20.87 ± 6.35 for T1-TSE, and 17.97 ± 6.83 for T2* according to the Berlin scoring system. Correlation with CT was strong for T1-TSE (r = 0.790) and very strong for T2* (r = 0.822). Inter-rater reliability was good for CT (ICC = 0.735, 95% CI: 0.539–0.848, p = 0.001) and excellent for both T1-TSE (ICC = 0.771, 95% CI: 0.605–0.867, p < 0.001) and T2* (ICC = 0.854, 95% CI: 0.746–0.915, p < 0.001). The diagnostic accuracy of JSC is shown in **Figure 3**.

#### 3.4.1 Ankylosis

Ankylosis was scored only in the SPARCC scoring system, and the mean total ankylosis score was 1.23 ± 3.27 for CT, 2.96 ± 3.04 for T1-TSE, and 0.63 ± 2.62 for T2*. Correlation with CT was strong for T1-TSE (*r* = 0.680) and very strong for T2* (*r* = 0.863). Inter-rater reliability was found to be excellent for CT (ICC = 0.944, 95% CI: 0.902–0.968, *p* < 0.001), fair for T1-TSE (ICC = 0.465, 95% CI: 0.078–0.690, *p* = 0.012), and excellent for T2* (ICC = 0.971, 95% CI: 0.950–0.983, *p* < 0.001).

Diagnostic performance metrics are presented in **Figure 3**, and lesion sum scores, CT correlations, and inter-rater reliability are summarized in **Table 2**. For both readers, the intra-rater intraclass correlation coefficients (ICCs) for total structural change scores obtained from MRI and CT ranged from 0.80 to 1.00, indicating excellent reliability. Fat metaplasia and backfill, as defined in the SPARCC scoring system, were excluded from the MRI–CT comparison because these lesions are not among the structural changes detectable by CT.

**Table 2:** CT and MRI structural lesions sum scores, Pearson and Inter-rater ICC coefficients.

BERLIN Scores	Sum scores mean (SD)	Pearson coefficient	p value	Inter-rater ICC coefficient	95% CI	p value
Erosion T1-TSE Sclerosis JSC	6.91 (3.63) 3.71 (3.77) 20.87 (6.35)	0.646 0.758 0.790	<0.001 <0.001 <0.001	0.695 0.882 0.771	0.474-0.823 0.797-0.932 0.605-0.867	<0.001 <0.001 <0.001
Erosion T2* Sclerosis JSC	13.54 (8.27) 10.93 (8.99) 17.97 (6.83)	0.913 0.921 0.822	<0.001 <0.001 <0.001	0.924 0.930 0.854	0.868-0.956 0.879-0.960 0.746-0.915	<0.001 <0.001 <0.001
Erosion CT Sclerosis JSC	12.13 (8.13) 11.01 (7.59) 21.04 (6.62)	–	–	0.897 0.939 0.735	0.820-0.941 0.894-0.965 0.539-0.848	<0.001 <0.001 0.001
SPARCC Scores	Sum scores mean (SD)	Pearson coefficient	p value	Inter-rater ICC coefficient	95% CI	p value
T1-TSE Erosion Ankylosis	9.19 (4.43) 2.96(3.04)	0.597 0.680	<0.001 <0.001	0.785 0.465	0.630-0.875 0.078-0.690	<0.001 0.012
T2* Erosion Ankylosis	17.13 (9.17) 0.63 (2.62)	0.909 0.863	<0.001 <0.001	0.953 0.971	0.918-0.973 0.950-0.983	<0.001 <0.001
CT Erosion Ankylosis	15.36 (8.79) 1.23 (3.27)	–	–	0.942 0.944	0.899-0.967 0.902-0.968	<0.001 <0.001

CI, confidence interval; CT, computed tomography; ICC, intraclass correlation coefficient; SD, standard deviation; TSE, turbo spin echo.

These qualitative imaging patterns are illustrated in representative cases shown in **Figure 2**, including concordant findings across modalities.

## 4 Discussion

First-echo T2* images reliably detected structural lesions, particularly erosions, with substantial inter-rater agreement. The combined use of the Berlin and SPARCC scoring systems also provided complementary assessment of lesion burden and distribution, strengthening the interpretation of our findings.

First-echo T2* images correlated more strongly with CT than conventional T1-TSE imaging for erosions, sclerosis, joint-space changes, and ankylosis, and consistently showed excellent inter- and intra-rater reliability (ICC > 0.8). These findings indicate that first-echo T2* imaging can provide a largely CT-concordant assessment of cortical and subchondral bone integrity without ionizing radiation. Recent studies indicate that bone marrow edema alone lacks specificity, as it can occur with mechanical loading and even in healthy or postpartum individuals. In contrast, erosions and other structural lesions are less common in non-axSpA populations and thus remain diagnostically useful. This highlights the importance of MRI protocols that reliably assess not only active inflammation but also cortical disruption, sclerosis, and joint-space changes.

Conventional T1-TSE imaging remains important for anatomical assessment and for identifying fat-containing structural lesions. However, its sensitivity for detecting small erosions is limited, and CT-referenced studies have reported sensitivities of 61% to 79% and specificities of 93% to 95% for erosion detection (17, 28, 29). To overcome this limitation, several gradient-echo–based techniques, including LAVA, SWI, UTE, VIBE, and ZTE, have been introduced to enhance cortical bone visualization in the SIJs (17, 20, 29–32). Thin-slice 3D gradient-echo techniques have demonstrated higher sensitivity than conventional T1-TSE imaging, although they are not yet widely available in routine practice.

In a study by Li et al. evaluating structural changes in the SIJs of 40 axSpA patients, T1-TSE and ZTE sequences were compared using CT as the reference standard. The ZTE sequence demonstrated higher sensitivity than T1-TSE for detecting erosions (98.2% vs. 80.0%) and sclerosis (89.5% vs. 40.4%), while their specificities were similar. Regarding joint space alterations, the sensitivities were comparable; however, ZTE showed higher specificity (93.0% vs. 67.4%) (32).

In a study by Lin et al. evaluating the diagnostic performance of ZTE and LAVA Flex (a VIBE-equivalent MRI sequence) sequences in the SIJs of 53 axSpA patients using CT as the reference standard, the ZTE sequence demonstrated higher sensitivity than the LAVA Flex sequence for detecting erosions and sclerosis (92.5% vs. 81.5% for erosions and 90.6% vs. 71.2% for sclerosis, respectively), whereas no significant difference was observed regarding joint space alterations. In this study, the performances of both sequences were not compared with that of the T1-FSE sequence (33).

In a study by Diekhoff et al. evaluating the diagnostic performance of SIJ T1-TSE and VIBE MRI sequences for the detection of erosions in 110 patients with low back pain referred for suspected axSpA, using CT as the reference standard, the sensitivity of the VIBE was found to be 93%, whereas the sensitivity of the T1-TSE sequence was 79%. The specificity was calculated as 93% for both sequences (29).

In the retrospective study by Baraliakos et al., T1-TSE, VIBE MRI, and CT images of 109 patients with axSpA were evaluated for the presence or absence of structural damage at the SIJ quadrant level. VIBE demonstrated a sensitivity of 71.2% and a specificity of 87.3% for the detection of erosions (34).

Deppe et al. compared the diagnostic performances of SWI and T1-TSE sequences for detecting erosions, sclerosis, and joint space alterations in the SIJs using CT as the reference standard. In this study including 68 axSpA patients, the sensitivities of SWI and T1-TSE for the detection of erosions were 81.6% and 73.7%, respectively, whereas their sensitivities for detecting sclerosis were 74.6% and 23.9%, respectively. No differences were detected regarding joint space alterations (30).

Ren et al. investigated the diagnostic performance of the 3D-UTE MRI sequence for the detection of SIJ erosions using CT as the reference standard. In this prospective study including 32 patients with axSpA, the presence of erosions at the SIJ quadrant level was independently evaluated by two readers. The 3D-UTE sequence demonstrated sensitivities of 94.7% and 92.9%, specificities of 97.4% and 96.5%, positive predictive values of 96.7% and 95.4%, negative predictive values of 95.9% and 94.5%, and accuracies of 96.2% and 94.9% for the two readers, respectively (35).

In the study by Hahn et al., which included 14 patients with axial spondyloarthritis, the correlation between UTE imaging and CT for the detection of SIJ erosions was reported as r = 0.70, 0.72, and 0.67 for the three readers, respectively. The sensitivities were 0.79, 0.86, and 0.71, while the specificities were 0.86, 0.93, and 0.79 for the three readers, respectively (31).

In our study, structural evaluation was based on short-TE first-echo images obtained from a multi-echo T2* mapping scan rather than dedicated 3D or thin-slice protocols. Despite this, erosion detection sensitivity reached 88.6%, which falls within the range reported for previous gradient-echo–based methods. Because CT served as the reference standard, first-echo T2*-positive/CT-negative findings were classified as false positives in the diagnostic performance analysis. Secondary re-review of the CT source semicoronal images in these discordant cases did not confirm corresponding cortical erosions. These discordant findings may partly reflect susceptibility-related accentuation of cortical margins, potentially mimicking cortical breaks and contributing to its lower specificity.

while Taken together with the high interobserver agreement for patient-level T2* erosion observed in this study (κ = 0.805), these findings support the feasibility of first-echo T2* imaging as a practical, radiation-free, and more accessible method for structural SIJ assessment.

A further advantage of this approach is its practicality. First-echo T2* images are generated directly from a sequence available on standard clinical MRI systems and do not require ultra-short echo times, thin-slice 3D acquisitions, synthetic CT reconstruction, or specialized post-processing. This may facilitate broader use outside specialized imaging centers.

This study has several limitations. First, the sample size was moderate and included patients at various disease stages, which may limit the generalizability of the findings. Second, the small number of patients in the non-axSpA subgroup (n=9) limits the precision of the specificity estimates and may reduce the generalizability of the diagnostic performance observed in this cohort. Therefore, these findings should be validated in larger cohorts with a more balanced representation of axSpA and non-axSpA patients. Third, given the low prevalence and limited burden of ankylosis in our cohort, reliability estimates for ankylosis should be interpreted with caution, as the limited number and variability of ankylotic lesions may have affected the stability of the ICC estimates. Fourth, although MRI and CT were performed within the same week in most patients, two patients had longer intervals of up to 12 weeks between examinations; therefore, interval changes in structural lesions cannot be completely excluded in these cases. Fifth, only a single echo time (TE = 4.36 ms) was analyzed, and the impact of TE optimization on diagnostic performance remains unknown. Sixth, we did not perform a direct comparison with thin-slice 3D gradient-echo sequences currently recommended by the ASAS–SPARTAN consensus for erosion-sensitive imaging. Such a comparison would better determine the relative diagnostic value of first-echo T2* images. Seventh, first-echo T2* images were not directly compared with the 2D fat-suppressed T1-weighted sequence that can be used when 3D acquisitions are unavailable. Eighth, CT was performed with a standard diagnostic protocol rather than a dedicated low-dose protocol; future validation studies should use low-dose CT as the reference standard. Ninth, the vendor default T2* mapping protocol was used without optimization of flip angle or repetition time; the first-echo images therefore have mixed contrast, and the effect of these parameters on lesion depiction was not evaluated. Furthermore, all MRI examinations were performed on a single vendor’s 3-Tesla system. Consequently, the diagnostic performance of the T2* sequence reported herein may not be directly applicable to MRI platforms from other manufacturers or to systems with different field strengths.

## 5 Conclusion

Short-TE first-echo images obtained from a multi-echo T2* mapping sequence demonstrated good agreement with CT for the detection of structural SIJ lesions, particularly for erosion assessment. By leveraging the inherent contrast characteristics of gradient-echo imaging, this approach may facilitate visualization of cortical and subchondral bone abnormalities without the need for dedicated ultra-short echo time acquisitions, thin-slice 3D imaging, or extensive post-processing. Given its availability on standard MRI systems, this technique may represent a promising adjunct for structural SIJ assessment. These findings should be interpreted in light of the study’s single-center design and limited sample size, and warrant further validation in larger, multicenter, multi-vendor cohorts before broader clinical implementation.

## Data Availability

The original contributions presented in the study are included in the article/**Supplementary Material**. Further inquiries can be directed to the corresponding author.
