# Supplementary material for: Short echo time first-echo images of T2* mapping for detecting structural sacroiliac joint lesions in axial spondyloarthritis: a CT-referenced study
Source: Front Immunol. 2026 Oct 22;17:1946946. doi: 10.3389/fimmu.2026.1946946 (PMC13736754; doi:10.3389/fimmu.2026.1946946)
Supplement: Supplementary file 1 [file Table1.docx]

**Supplementary Table 1.** Magnetic resonance imaging sequence parameters.

T1-TSE, T1-weighted turbo spin-echo; STIR, short tau inversion recovery; TR, repetition time; TE, echo time; ms, milliseconds; mm, millimeters; cm, centimeters; kHz, kilohertz.

| **Sequence** | **Repetition Time (TR)** | **Echo Time (TE)** | **Slice Thickness** | **Matrix** | **Flip Angle** | **Field of View** | **Bandwidth** |
| --- | --- | --- | --- | --- | --- | --- | --- |
| T1-TSE | 449 ms | 10 ms | 3 mm | 448 × 336 | 131° | 23 cm | 248 kHz |
| T2* mapping | 784 ms | 4.36, 11.90, 19.44, 26.98, 34.52 ms | 3 mm | 512 × 512 | 60° | 34 cm | 257 kHz |
| STIR | 6130 ms | 49 ms (Inversion Time = 210 ms) | 3 mm | 512 × 512 | 155° | 24 cm | 200 kHz |
