# Supplementary material for: Short echo time first-echo images of T2* mapping for detecting structural sacroiliac joint lesions in axial spondyloarthritis: a CT-referenced study
Source: Front Immunol. 2026 Oct 22;17:1946946. doi: 10.3389/fimmu.2026.1946946 (PMC13736754; doi:10.3389/fimmu.2026.1946946)
Supplement: Supplementary file 2 [file Table2.docx]

**Supplementary Table 2** Computed tomography acquisition parameters and radiation dose metrics.

| **Tube Voltage** | **Tube Current** | **Gantry Rotation Time** | **Pitch Factor** | **Slice Thickness** |
| --- | --- | --- | --- | --- |
| 120 kVp | Automatic exposure control | 0.8 s | 0.98 | 0.63 mm |

CT, computed tomography; kVp, kilovolt peak; s, seconds; mm, millimeters.
