# Supplementary material for: Short echo time first-echo images of T2* mapping for detecting structural sacroiliac joint lesions in axial spondyloarthritis: a CT-referenced study
Source: Front Immunol. 2026 Oct 22;17:1946946. doi: 10.3389/fimmu.2026.1946946 (PMC13736754; doi:10.3389/fimmu.2026.1946946)
Supplement: Supplementary file 3 [file Table3.docx]

**Supplementary Table 3.** Radiation dose metrics for sacroiliac joint CT examinations (n = 54).

| **Metric** | **Summary** | **Range** |
| --- | --- | --- |
| CTDIvol, mGy | 14.35 (9.73–18.75) | 4.50–27.66 |
| Total DLP, mGy·cm | 449.0 ± 165.5 | 127.8–828.5 |
| Estimated effective dose, mSv | 6.42 ± 2.37 | 1.83–11.85 |

CTDIvol is presented as median (interquartile range), whereas total DLP and estimated effective dose are presented as mean ± standard deviation. All dose metrics were referenced to a 32-cm body phantom. Estimated effective dose was calculated as total DLP × 0.0143 mSv/(mGy·cm), using the adult pelvis-specific conversion coefficient reported by Huda et al. [35]. Abbreviations: CT, computed tomography; CTDIvol, volume CT dose index; DLP, dose-length product; mGy, milligray; mSv, millisievert; cm, centimeter.
